# Symptomatic Imperforate Hymen in Early Infancy: A Case Report

**DOI:** 10.31729/jnma.4922

**Published:** 2020-06

**Authors:** Geha Raj Dahal, Subash Phuyal, Pooja Agrawal

**Affiliations:** 1 Department of Pediatric Surgery, Tribhuwan University Teaching Hospital, Kathmandu, Nepal; 2 Department of Radiology, Grande International Hospital, Dhapasi, Kathmandu, Nepal; 3 Department of Radiology, Norvic International Hospital, Thapathali, Kathmandu, Nepal

**Keywords:** *hydrocolpos*, *hymen*, *mullerian ducts*

## Abstract

Imperforate hymen, though a congenital anomaly, usually presents late in puberty as lower abdominal pain, primary amenorrhea, and cyclical pain. Blood collects in vagina and uterus, proximal to imperforate hymen leading to their distention. Its presentation at infancy is a rare entity. We report such a rare case of symptomatic imperforate hymen in infancy, who presented with acute retention of urine, chills and rigor. Abdominal examination revealed an intra-abdominal mass in the lower abdomen and pelvis with the absence of vaginal opening on perineal examination. Contrast-enhanced computed tomography abdomen showed large abdominopelvic cystic lesion posterior to the urinary bladder and anterior to the rectum consistent with a highly distended vagina. She was managed by the incision of the imperforate hymen and drainage of the pus. A high index of suspicion is necessary whenever a female infant presents with abdomino-pelvic mass with symptoms of fever or urinary retention.

## INTRODUCTION

Imperforate hymen is an uncommon congenital disorder where there is complete obstruction of the vaginal opening by the hymen. The worldwide incidence is estimated at 0.014-0.1%. It is usually asymptomatic until menarche with an average presentation age of 11-15 years. It is rarely diagnosed in the neonatal period.^1^ At puberty, they typically present with vaginal bulge caused by hematocolpos behind it.^2^ Before puberty, imperforate hymen rarely leads to hydrocolpos or mucocolpos and if infected pyocolpos, which may again rarely cause obstructive uropathy through the compression of the lower urinary tract, resulting in hydronephrosis, hydroureters and subsequently renal

failure.^3^

## CASE REPORT

A 4-month-old female infant presented with acute retention of urine and chills and rigor for 2 days. On examination, she was of normal weight and height for age. General physical examination was normal except for pyrexia. An abdominal examination revealed an intra-abdominal mass-like lesion in the lower abdomen and pelvis. There was no vaginal opening on perineal examination. The urethral and anal opening was normal. No other abnormalities were detected on systemic review.

Contrast-enhanced computed tomography (CECT) abdomen showed large well-defined abdominopelvic cystic lesion posterior to the urinary bladder and anterior to the rectum in the rectovesical pouch; measuring about 14.0cm × 6.7cm × 6.3cm (craniocaudal (CC) × transverse (TR) × anteroposterior (AP)) consistent with a highly distended vagina. Superiorly it was communicating with a cystic tubular structure, which represented displaced and mildly distended uterus.

**Figure 1:**
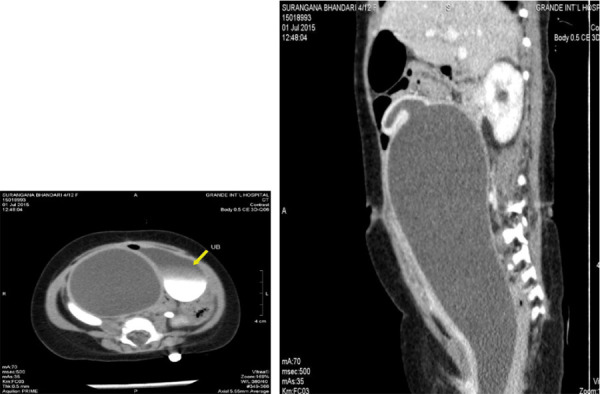
Pre-operative CECT abdomen. Large well-defined abdominopelvic cystic lesion is posterior to the urinary bladder and anterior to the rectum in the rectovesical pouch c/w highly distended vagina. Superiorly it is communicating with a cystic tubular structure likely displaced uterus.

Bilateral hydronephrosis and proximal hydroureter are also observed (Figure 1). The thickness of hymen was 4.3mm on ultrasonography.

**Figure 2:**
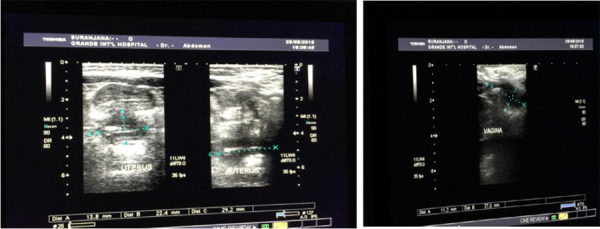
Post-operative USG. Postoperative ultrasonography (after 6 weeks) of the pelvic region revealed a normal size uterus with a small amount of fluid (27 × 4 mm) within the vagina.

The cruciate incision was made on hymen. Approximately 500ml of foul-smelling pus was drained. The postoperative period was uneventful. The patient was discharged on postoperative day 2. On 6 weeks follow up, the vagina and uterus regressed in size by ultrasonography (Figure 2). The opening of the hymen was patent.

## DISCUSSION

The hymen is a membranous structure lying between the vagina and the vulva. Embryologically, it corresponds to the junction of the caudal end of the Mullerian duct which forms the upper 2/3^rd^ of the vagina and the urogenital sinus which forms the lower 1/3^rd^. Usually, hymen ruptures before birth due to degeneration of central epithelial cells.^4^ Imperforate hymen occurs due to abnormalities in the degeneration of the central epithelial cells. This may result in hydrocolpos which is cystic dilation of the vagina with fluid accumulation proximal to obstruction due to stimulation of secretory glands of the reproductive tract and maternal estrogen.^5^ On examination of the genitalia of a newborn, it can be appreciated as a bulging membrane. Following this, there is the reabsorption of the mucus and the child becomes asymptomatic only to present with various symptoms after menarches such as primary amenorrhea, cyclical abdominal pain or urinary retention.^6^

The complications of congenital hydrocolpos include urinary tract obstruction due to compression, renal failure, repeated urinary tract infections, rupture, and peritonitis. Sepsis leading to death can occur secondary to either urinary tract infection or rupture and secondary peritonitis.^7^,^8^

These complications can be managed by simple hymen incision and drainage of the collection. Therefore, a thorough newborn examination is essential to screen for an imperforate hymen. Magnetic resonance imaging is especially helpful when hydrometrocolpos is secondary to cloacal malformation. Magnetic resonance imaging is also preferred when ultrasonography is limited by the patient’s obesity or oligohydramnios in suspicious antenatal scans.^7^

Neonatal hydrocolposis a rare condition and occurs secondary to vaginal obstruction and stimulated cervicovaginal secretions. A high index of suspicion is necessary whenever a female infant patient presents with abdomino-pelvic mass with symptoms of fever or urinary retention. Diagnosis can be confirmed using ultrasonography, computed tomography, or magnetic resonance imaging, with MRI being of particular use in the presence of other cloacal abnormalities due to its superior soft-tissue resolution. Early diagnosis reduces the incidence of complications such as infection, rupture, and renal failure.

**Consent: JNMA** Case Report Consent Form was signed by the patient and the original is attached with the patient chart.

## Conflict of Interest:

**None.**
